# The Prevailing Excitement

**Published:** 1874-04

**Authors:** 


ELMIRA, N. Y., APRIL, 1874.

The BISTOURY is published Quarterly, upon the 1st of April, July, October and January, at Fifty Cents a year, in advance.

### THE PREVAILING EXCITEMENT.

The Americans are, notably, a people fond of "sensations." They are restless under restraint, and, consequently on the *qui vive* for something to transpire which may afford them the necessary excitement, to which they may devote their unemployed moments. Just now, this peculiarity is abundantly gratified in the advent of a new phase of the old question of temperance. Having exhausted the fund of amusement, or excitement, found in defending the Maine Liquor Law, Local Option and the like, and in attending upon the conclaves of the various mystic societies, founded for the promulgation and advancement of temperance principles, a new phase of the question has suddenly burst upon us,—just in the nick o' time, to save the entire temperance question from being sunk in oblivion. And this time we are indebted, it appears, to Dr. Dio Lewis, for giving us a *real* sensation, in inducing the women,—those modest, timid and lovely creatures, to throw off those womanly traits of character, for which they have been so long loved and admired, and appear upon our public thoroughfares blossomed into genuine and earnest reformers.

All this is prefatory to asserting that we are opposed to the movement, as now conducted,—opposed to the ostentatious, sensational, and public manner, in which it is proposed to exhibit our wives and daughters. We believe they are the proper persons to manage and execute such a reform, but not after the formula prescribed by Dr. Lewis.

As this statement exposes us to the risk of censure from temperance people, many of whom we greatly respect and admire for their earnestness and self-sacrificing devotion to a much needed reform, we will give a necessarily brief outline of our reasons for such opposition :—

1.—The prayers are not offered at the proper doors ! This assertion we support with the statement that, the saloonkeeper is engaged in a legitimate and popularly recognized pursuit, protected by the Government, for which he pays a tax,—a larger one than any other trader. Now, as long as he *is* so protected, and so long as he obeys the laws regulating such traffic (in the matter of not selling intoxicating drinks to minors, or to habitual drunkards,) no man nor woman, has *any* right whatever, to intefere with his business affairs, by trespassing upon his premises, either for the purpose of prayer, or other devotional exercises. Aside from this consideration, it is unjust, unfair, and presumptious, upon the part of any ladies, to ask such a man to close his saloon, destroy his liquors, and abandon his vocation, entirely at his own loss and expense !

If, with such a proposition, came offers of pecuniary remuneration, as well as prayers, perhaps the saloon keeper would be more deeply impressed with the earnestness of his fair visitors ; for, after all, a noteworthy measurement of man or woman's devotion to any cause, calculated to benefit their race, is to consider the amount in currency, invested with their zeal and prayers.

This is not offered in extenuation of the sins of the liquor-seller, nor as evidence of our approval of his vocation. It is not one that we would select for any member of our family, nor recommend to a friend ; yet, it *is* a business, recognized by our laws, and as such is entitled to our protection, if not our admiration.

Where then *should* these prayers be offered ? In the dwellings of, and with the miserable drunkards ; praying that they may desist from this health and soul destroying vice ; and, through the gentle, kind and ennobling influences of women, persuade them to abandon the debasing habit —seek to reform them by prayers, pecuniary assistance—by restoring them to the employment which became forfeited through inebriety—then, keep them there, by constant watchfulness and care. We believe that woman, by her gentle, sympathizing, and beseeching efforts, can accomplish more good at *this* door, than by the ostentatious display of these same powers, upon our street corners.

The next doors that require visitation, is the ones behind which reside our member of Assembly and our honorable member of Congress. Pray with *them*, ladies,—pray that they so amend our liquor laws as to render it a penal offence, punishable by ten year's imprisonment, at hard labor, for any person to sell liquor to any minor, or to any man or woman, known to be a habitual drunkard. Ask also, that the tax upon all manner of liquor be advanced to from $10 to $20 a gallon,—make it so expensive, that the gentleman bummer will be required to pay one dollar for his "whiskey straight," instead of ten cents, as now. Make it so expensive a luxury that no habitual drunkard can earn enough in a week, to purchase a sufficient quantity of the fluid, to induce one respectable "drunk." And, while you are at it, ladies, pray for an amendment to our laws, that it shall become a costly offense for any one to offer for sale any but absolutely pure and unadulterated liquors. Let it be the duty of the inspecting officer to destroy all adulterated, drugged and impure liquors, wherever encountered, and report the individual in whose possession it is found, to the proper authorities, for severe and summary punishment; for, believe me, to the cheapness and vileness of our beverages, the major portion of all drunkenness is due.

Let your prayers incline in this direction, my dear ladies, for, when you have succeeded in securing such legislation, and then stand prepared and watchful, to see that it is strictly enforced, you will have accomplished more good than will accrue from the present spasmodic temperance movement.

Let me admonish you, ladies, that, while you are talking and praying temperance to those poor unfortunates who are in need of such advice, do not be intemperate yourselves; I mean in the matter of your zeal. Do not strive to outdo the members of a sister church in the good work, and so exhibit your own weakness. Strive to set a better example even, than do those ministers of the Gospel of Christ, who berate and even insult a brother minister, for expressing antagonistic views, with reference to this reformation. Such a procedure would be unworthy of any christian man or woman, and would greatly retract from your usefulness; for, believe me, those drunken men have sober, lucid intervals, in which they do much thinking, and in which their perceptive powers are so quickened, as to be able even to recognize these meagre idiosyncrasies, and they are quite likely to acquaint you with them, when you strive to inculcate them with a proper appreciation of the christian graces, perhaps to your chagrin and mortification. Be above reproach yourselves, then go forth in the good work, and may God bless your endeavors.

The prayers, we believe to be offered to the wrong persons, because they are not the *real* offenders. As long as there exists a *demand* for liquor, so long will men be found to abundantly supply the want. The saloonkeeper only acts as a medium between producer and consumer. If you persuade him to vacate his office, as "middle man," between the distiller and the consumer, you neither destroy the demand, nor suppress the supply. You only force the habitual bibler to purchase at wholesale of the distiller, that which he before secured in smaller quantities. Therefore, it seems clear to us that, the consumer first, who creates the demand—then the manufacturer, who supplies it, need most your prayers and womanly petitions.

Again, your prayers should be addressed to your own households, in the endeavor to create a wholesome, public sentiment, with reference to inebriety and wine bibling. Strive to make it unpopular to imbibe stimulants of all kinds, by forbidding your daughters to receive the attentions of young men so inclined; while you are careful that nothing of the sort is ever offered your friends, in your own homes. You can accomplish much in this direction, by concerted and unanimous action, so that it will be quite as unpopular for a young man to be found in the beer saloon, as in those dens of infamy, the names of which are never breathed in polite society. This, to us, appears so clear, as to seem a useless waste of words to further enlarge upon it.

2. Another objection to the present movement, and as strong a one perhaps, as the one just given, is that we doubt, indeed cannot bring ourself to believe, for an instant, that any permanent good will result from this excitement. It is strictly and emphatically a "sensation," a sort of revival, if you please, the success of which depends solely upon excitement and ostentation. It may sweep through our land like a whirl-wind, completely uprooting and upsetting every opposition that may be brought to bear upon it; and, the rejoicing thereat, will be great; so great, indeed, that the saloon keeper himself, will be swallowed in this unrelenting vortex, and imagine himself a popular object of curiosity and admiration, by reason of his self-sacrifice. But, when the calm, which always follows the storm, ensues, the saloon-keeper, I fear, will become restless, and long for his old traffic and associates; and, one by one, you will likely see the old, familiar signs, reappear; whereat, the ladies will become disheartened, and few will be found courageous enough to fight the lost battle over again.

3.—We oppose, because temperance, when *enforced*, is antagonistic to our ideas of self-government; and, therefore, can never succeed. We, as Americans, are proud of our independence,—our freedom to do pretty much as we please; and, when you attempt to curb such freedom of action, you at once arouse our ire, and enlist our opposition. Therefore, if you declare that we *shall* not drink at the dram-shop, nine-tenths of us *will* drink at our private homes, or at the club, if for nothing else than to exhibit our independence in the matter; and we firmly believe that not only every habitual drinker will have a private saloon of his own, but many will be added to the number from among those who rarely, or *never* drink, simply to exhibit to their friends their irrepressible proclivities.

In view of these facts, we again revert to our original proposition, viz: Your prayers are not offered at the proper doors! When they *are* made, in accordance with the ideas just advanced, we firmly believe that they will be answered with much good. And then, dear ladies, our prayers shall mingle with yours, with the consciousness too, of their availability:—"May the good Lord bless your endeavors to suppress the growing, and debasing evil, of intemperance."

